# Clinical and Genetic Analysis of Multiple Endocrine Neoplasia Type 1-Related Primary Hyperparathyroidism in Chinese

**DOI:** 10.1371/journal.pone.0166634

**Published:** 2016-11-15

**Authors:** Jing Kong, Ou Wang, Min Nie, Jie Shi, Yingying Hu, Yan Jiang, Mei Li, Weibo Xia, Xunwu Meng, Xiaoping Xing

**Affiliations:** 1 Department of Endocrinology, Key Laboratory of Endocrinology, National Health and Family Planning Commission, Peking Union Medical College Hospital, Chinese Academy of Medical Science & Peking Union Medical College, Beijing, People’s Republic of China; 2 Department of Pathology, Peking Union Medical College Hospital, Chinese Academy of Medical Sciences & Peking Union Medical College, Beijing, People’s Republic of China; 3 Department of Laboratory Medicine, Peking Union Medical College Hospital, Chinese Academy of Medical Science & Peking Union Medical College, Beijing, People’s Republic of China; Harvard Medical School, UNITED STATES

## Abstract

**Objective:**

Multiple endocrine neoplasia type 1-related primary hyperparathyroidism (MHPT) differs in many aspects from sporadic PHPT (SHPT). The aims of this study were to summarize the clinical features and genetic background of Chinese MHPT patients and compare the severity of the disease with those of SHPT.

**Design and Methods:**

A total of 40 MHPT (27 sporadic, 7 families) and 169 SHPT cases of Chinese descent were retrospectively analyzed. X-rays and ultrasound were used to assess the bone and urinary system. Dual energy x-ray absorptiometry (DXA) were performed to measure bone mineral density (BMD). Besides direct sequencing of the *MEN1* and *CDKN1B* genes, multiplex ligation-dependent probe amplification (MLPA) was used to screen gross deletion for the *MEN1* gene.

**Results:**

Compared with SHPT patients, MHPT patients showed lower prevalence of typical X-ray changes related to PHPT (26.3% vs. 55.7%, P = 0.001) but higher prevalence of urolithiasis/renal calcification (40.2% vs. 60.0%, P = 0.024). MHPT patients showed higher phosphate level (0.84 vs. 0.73mmol/L, P<0.05) but lower ALP (103.0 vs. 174.0U/L, P<0.001) and PTH (4.0 vs. 9.8×upper limit, P<0.001) levels than SHPT patients. There were no significant differences in BMD Z-scores at the lumbar spine and femoral neck between the two groups. Mutations in the *MEN1* gene were detected in 27 MHPT cases. Among the nine novel mutations were novel, one of them involved the deletion of exon 5 and 6.

**Conclusions:**

MHPT patients experienced more common kidney complications but less skeletal issues, and a milder biochemical manifestation compared with SHPT patients. *MEN1* mutation detection rate was 79.4% and 9 of the identified mutations were novel.

## Introduction

Primary hyperparathyroidism (PHPT) is a common endocrine disease characterized by simultaneously elevated serum calcium and parathyroid hormone (PTH) levels. The majority of cases are sporadic PHPT (SHPT), and less than 10% of cases are hereditary or part of a familial syndromes. Multiple endocrine neoplasia type 1 (MEN1, OMIM #131100) is an autosomal dominant inherited disease characterized by the occurrence of several endocrine tumors. MEN1 accounts for approximately 70% hereditary PHPT [1] and parathyroid tumors occur in nearly 95% of MEN1 patients [2].

The clinical manifestations of MEN1-related PHPT (MHPT) differ from SHPT in many aspects, including an earlier age of onset, more common multiglandular lesions, and milder biochemical presentation in MHPT patients [3, 4]. Although change of bone mineral density (BMD) has been widely evaluated in SHPT patients, information on BMD in MHPT patients are limited. Only Eller-Vainicher *et al*. had compared BMD between SHPT and MHPT patients, and their results suggested more severe bone loss in MHPT than in SHPT patients of Italian descent [3]. In addtion, the renal complications secondary to MHPT are seldom studied.

MEN1 is caused by germline mutations of *MEN1*, a tumor suppressor gene that encodes the menin protein [5]. More than 1300 germline and somatic *MEN1* mutations have been reported, without any obvious genotype-phenotype correlations [6]. However, MEN1 mutations could not be detected in approximately 5 to 20% of MEN1 patients and the negative detection rate was as high as 76% in a large Swedish study [2, 7]. The variability in *MEN1* mutation detection rates may partially be attributed to different detection methods, phenotype ascertainment or presence of mutations involved in other genes [2]. Moreover, it is necessary to investigate mutations in the *CDKN1B* gene because MEN4 exhibits phenotypes that overlap those of MEN1.

To date, little is known about the clinical spectrum and genetic background of MHPT patients in China [8, 9]. Therefore, we compared the clinical characteristics of MHPT to those of SHPT in Chinese patients using a relatively large sample size and included details on severity of the disease, biochemical parameters and radiographic results. The genetic background of these patients was investigated by regular PCR and multiplex ligation-dependent probe amplification assay (MLPA).

## Materials and Methods

### Ethics statement

The present study was approved by the Institution Review Board (IRB) of Peking Union Medical College Hospital (No. S-K 106), and was exempted from full IRB review as this retrospective study only utilizes existing data and pathological specimens that are publicly available. Written informed content was obtained from all subjects or parents of subjects under 18 years old and healthy controls. All clinical investigations were conducted according to the principles documented in the Declaration of Helsinki. Written informed consent (as outlined in PLOS consent form) to publish details of their cases were obtained from the patients or their parents.

### Subjects and Clinical investigation

From February 2002 to February 2013, 686 PHPT patients including 55 (8.0%) MHPT patients were diagnosed in Peking Union Medical College Hospital. A total of 40 consecutive MHPT patients with detailed follow-up data, who have provided consent to genetic analysis, were included in the present study. To reduce random and systematic bias, a total of 169 SHPT patients were randomly selected from all the PHPT patients that were followed up for at least one year and showed no recurrent hypercalcemia during the same period. The authors were given access to participant- identifiable information during and after data collection due to the retrospective nature of the study and convenience for collecting additional family information.

Diagnosis of PHPT was defined biochemically as serum calcium (SCa) level greater than 2.70 mmol/L and/or serum ionized calcium (iCa) greater than 1.28 mmol/L combined with unsuppressed PTH level. Patients with familial benign hypocalciuric hypercalcaemia (FHH) were excluded [10]. Asymptomatic PHPT is defined as hyperparathyroidism that lacks specific symptoms or signs traditionally associated with hypercalcemia or PTH excess. The presence of MEN1 syndrome was evaluated in every PHPT patient based on full personal and family history, along with laboratory and imaging evaluation of pancreas, pituitary, and adrenal glands. MEN1 syndrome was confirmed by one of three criteria [2, 11]: (1) presence of two or more major MEN1-associated endocrine tumors (i.e. parathyroid adenoma, enteropancreatic tumor, and pituitary adenoma); (2) presence of one of the MEN1-associated tumors in a first-degree relative that was clinically diagnosed with MEN1; and (3) identification of a germline *MEN1* mutation, who might be asymptomatic and has not developed biochemical or radiological manifestations of MEN1. Relatives of MEN1 probands were screened for MEN1 syndrome with their consent.

Preoperative localization including ultrasonography and 99m-sestamibi-scintigraphy (MIBI) was conducted in all PHPT patients. Typical X-rays features of PHPT, including subperiosteal absorption, osteitis fibrosa cystic and osteomalacia, were analyzed. BMD was measured by DXA (GE-Lunar, USA) at the lumbar spine (L2-L4, CV: 1.70%) and femoral neck (FN, CV: 1.73%) in 104 SHPT and 35 MHPT patients. Z-scores and T-scores of BMD were calculated using database on normal subjects in our center [12]. Z-scores lower than -2.0 were compatible with reduced BMD. Urolithiasis or renal calcification was assessed by ultrasound.

### DNA isolation and direct sequencing

Genomic DNA was extracted from peripheral blood lymphocytes of MHPT patients, their family members and 100 healthy Chinese controls using the QIAamp DNA blood mini kit (Qiagen, Germany). All coding exons and exon-intron boundries of the *MEN1* gene were amplified by PCR using DNA from the probands. If no mutation was found in the *MEN1* gene, the *CDKN1B* gene was screened. The regions of interest were amplified in other family members. The primers were designed by the software Oligo 7 (S1 Table). Direct sequencing of PCR products was performed using a TaqBig Dye terminator sequencing kit and an ABI3730 automated sequencer (Applied Biosystems, USA). PCR products were subjected to direct sequencing.

### Multiplex ligation-dependent probe amplification (MLPA)

For those patients with no *MEN1* or *CDKN1B* gene mutation as detected by routine PCR, MLPA was further performed to screen for large deletions in the *MEN1* gene using the SALSA MLPA probemix P017-C1 *MEN1* kit (MRC-Holland, Amsterdam, Netherlands) according to the manufacturer’s instructions. PCR products were analyzed on an AB3730 XL capillary electrophoresis apparatus (Applied Biosystems, USA). Dosage quotients (DQs) were calculated by comparing peak height values for each sample to those of three normal controls. The data were analyzed using GeneMarker 1.75 software. Ordinary PCR was used to detect the breakpoints of gross deletion detected by MLPA. Primers across exon 4–7 of the *MEN1* gene were designed to located the deletion sites by Sanger sequencing of the PCR products (S1 Table).

### *In Silico* prediction of variant pathogenicity

The predicted effects of mutations on *MEN1* gene were assessed using Polyphen-2 (http://genetics.bwh.harvard.edu/pph2/), SIFT (http://sift.jcvi.org/www/SIFT_enst_submit.html), and Mutation Taster (http://www.mutationtaster.org) software.

### Tissue samples and menin immunohistochemistry (IHC)

Thirty-one paraffin-embedded parathyroid tumor tissues from MHPT patients were available for IHC analysis. Six normal parathyroid glands incidentally removed during thyroid surgery for hyperthyroidism patients without clinical and biochemical evidence of PHPT were used as controls. IHC was performed for menin using a rabbit polyclonal antibody (Abcam plc, ab2605, Cambridge, UK; 1:300). All samples were independently tested in triplicate. Cells were scored as positive if specific nuclear staining was detected. Staining was quantified as the percentage of positive cells independent of the intensity of staining: less than 5% of positive cells as (–), 5~50% of positive cells as (+), 50~95% of positive cells as (++), and over 95% of positive cells as (+++). Two blinded pathologists were asked to evaluate each section independently. Slides were evaluated under a Nikon Eclipse 80i microscope (Nikon, Japan).

### Genotype-phenotype correlation analysis

In order to uncover genotype—phenotype correlation, mutation sites and types of mutation were taken into account when comparing the difference in clinical presentation, biochemical parameters, pathologic findings and follow-up outcomes. The subjects in group 1 carried nonsense, frameshift, gross deletion, and splice site mutations. These mutations are predicted to result in either a truncated menin protein or a loss of the translated protein due to nonsense-mediated mRNA decay (NMD),. On the other hand, subjects in group 2 carried missense mutations.

### Statistical analysis

Statistical analysis was performed using the SPSS 17.0 version statistical package (SPSS, Chicago, USA). The normally distributed data were expressed as mean ± SD, while median (inter-quartile range) was used for non-normal variables. Between-group differences were analyzed using independent-samples t test or One-Way ANOVA for normally distributed data, while Mann-Whitney U test was performed for the non-normal continuous variables. Categorical variables were compared by Pearson χ^2^ test, Fisher exact test or continuity-adjusted χ^2^ test. Multivariate analysis was tested by Logistic regression analysis or analysis of covariance. Missing data were removed from the numerator and the denominator simultaneously when calculating the rates or percentage. P < 0.05 was considered as statistically significant.

## Results

### Clinical data

The clinical characteristics of 40 MHPT patients and the comparison with SHPT patients are summarized in Table 1. Among them, 27 patients showed no family history of MEN1 (sporadic MHPT) while 13 patients were from 7 MEN1 families (familial MHPT). Urolithiasis/renal calcification was present in 24 patients (60.0%) and was the first clinical manifestation of PHPT in 18 MHPT patients (45.0%). Eight patients in the MHPT group (20.0%, 8/40) and 16 patients in the SHPT group (9.8%, 16/163) were confirmed as asymptomatic PHPT.

**Table 1:** Clinical characteristics of patients with MHPT and SHPT.

	MHPT (n = 40)	SHPT (n = 169)	P	P^#^
Sex (M/F)	13/27	48/121	0.746	
Age* (years)	45.0±14.0	50.7±14.6	0.025	
Course of PHPT (years)	7 (7)	3(5)	<0.001	
Typical changes in X-rays^§^	26.3%(10/38)	55.7%(83/149)	0.001	0.005
Gastrointestinal symptoms	32.5%(13/40)	43.2%(73/169)	0.216	0.515
Urolithiasis/renal calcification	60.0%(24/40)	40.2%(68/169)	0.024	0.045
Asymptomatic PHPT	20.0% (8/40)	9.8% (16/163)	0.130	0.690
SCa (mmol/L)	2.89±0.23	2.96±0.36	0.227	0.397
iCa (mmol/L)	1.41±0.20	1.49±0.23	0.062	0.199
P (mmol/L)	0.84±0.14	0.73±0.18	<0.001	<0.001
ALP (U/L)	103.0(67.0)	174.0(452.2)	<0.001	0.006
PTH (×UL)	4.0(4.7)	9.8(17.6)	<0.001	0.001
Cr (μmol/L)	74.1±28.1	85.6±39.3	0.085	0.244
UCa (mmol/24h)	9.76±4.62	10.20±5.20	0.630	0.810
UP (mmol/24h)	18.44±6.73	21.57±14.57	0.206	0.390
LS BMD (Z-score)	-1.693±1.403	-1.665±1.616	0.928	0.829
FN BMD (Z-score)	-1.601±1.187	-1.691±1.513	0.749	0.510
LS BMD (T-score)	-2.220±1.439	-2.531±1.551	0.297	0.758
FN BMD (T-score)	-1.964±0.793	-2.302±1.139	0.106	0.435

^#^, P value adjusted for age, sex and course of PHPT.

*, the age at diagnosis of PHPT.

Data are expressed as mean ± SD, median (inter-quartile range) or as percentage.

^§^, typical changes of PHPT in X-ray including subperiosteal absorption, osteitis fibrosa cystic and osteomalacia.

PHPT: primary hyperparathyroidism. MHPT: multiple endocrine neoplasia type 1-related PHPT. F: female; M: male; SCa: serum total calcium. iCa: serum ionized calcium. P, serum phosphorous. ALP, alkaline phosphatase. PTH, serum intact parathyroid hormone. UL: upper limit. Cr, creatinine. UCa, 24h urinary calcium. UP, 24h urinary phosphorous. BMD, bone mineral density. LS, lumbar spine. FN, femoral neck.

The mean age at diagnosis of PHPT in the MHPT group was younger than that in the SHPT group. Urolithiasis/renal calcification presented more frequently in MHPT patients (60.0% vs. 40.2%, P = 0.024), a difference that remained after adjusting for age, sex and course of PHPT, (P = 0.045). Among the biochemical parameters, decline of serum phosphate (P) level was less severe in the MHPT than in SHPT group (P<0.001). ALP (alkaline phosphatase) and PTH levels were lower in MHPT group than those in the SHPT group (both P<0.001). The differences remained after adjusting for sex, age and course of PHPT. Moreover, only one MHPT patients showed both of SCa and iCa levels to be within the normal range.

Typical X-rays features of PHPT was less common in MHPT patients compared to SHPT patients even after adjusting for age, sex and course of PHPT (26.3% vs. 55.7%, P = 0.001). However, there were no significant differences in the Z-scores of LS and FN between the two groups, even after adjustment of age, sex, course of PHPT and involvement of pituitary gland, pancreas, and adrenal glands (LS: P = 0.353, FN: P = 0.482). Among the 35 MHPT patients, 17 (48.6%) exhibited reduced BMD (Z<-2.0) at the LS and 9 (25.7%) exhibited reduced BMD (Z<-2.0) at the FN. Fractures were reported and verified in 12 MHPT patients (30%), whose Z-scores of LS and FN were both less than those patients without fractures (LS: -2.865±0.920 vs. -1.156±1.258, P<0.001; FN: -2.391±1.618 vs. -1.239±0.714, P = 0.006). PTH level correlated inversely with Z-scores of LS and FN in MHPT patients (R^2^: 0.181, 0.240, respectively; P: 0.011, 0.003, respectively). However, correlations between SCa level and Z-scores of LS and FN were absent in MHPT subjects (R^2^: 0.004, 0.003 respectively; P: 0.714, 0.747, respectively). Analysis of the SHPT group supported similar results that PTH, rather than SCa, correlated with BMD (data not shown).

### Genetic analysis of *MEN1* gene

Germline *MEN1* gene mutations were identified in the seven referred families (7/7, 100%). Nineteen different germline *MEN1* gene mutations were detected in 20 sporadic MHPT patients (20/27, 74.1%) (Table 2). *CDKN1B* gene mutation was not identified in this series of MHPT patients. In total, 27 MHPT patients were found to harbor *MEN1* mutations including seven missense mutations (7/27, 25.9%), four nonsense mutations (4/27, 14.8%), seven splice site mutations (7/27, 25.9%), eight frameshift mutations (8/27, 29.6%), and one gross deletion mutation (1/27, 3.7%). The 7 missense mutations led to single amino acid change (7/27, 25.9%), while the other 20 mutations were predicted to result in truncated protein (20/27, 74.1%). No significant differences in the clinical manifestations, biochemical parameters and pathology types were found between patients with missense mutations and those with mutations resulting in truncated protein (data not shown). Mutations in exon 2 presented more frequently than those in other exons (7/27, 26%), but hot spot mutation region was not observed.

**Table 2:** Mutations of *MEN1* gene in studied MHPT patients.

Case no.	Nucleotide alteration	Location	Mutation type	Reference
**F1**	c.654+1 G>T	Intron 3	Splice site	Cardinal, 2005, J Med Gnet
**F2**	c.1606 C>T (p.Q536X)	Exon 10	Nonsense	Wautot, 2002, Hum Mutat
**F3**	c.783+116_c.913-296del774	Exon 5,6	Gross deletion	**Novel**
**F4**	c.249_252delGTCT	Exon 2	Frameshift	Klein, 2005, Genet Med
**F5**	c.631_634delGTCA	Exon 3	Frameshift	Asteria, 2001, Hum Mutat
**F6**	c.457 G>T (p.D153Y)	Exon 3	Missense	**Novel**
**F7**	c.124 G>A (p.G42S)	Exon 2	Missense	Sato,2000, Surgery
**P14**	c.839_840 insT	Exon 6	Frameshift	**Novel**
**P15**	c.512 G>A (p. R171Q)	Exon 3	Missense	Balogh, 2007, Clin Endocrinol
**P16**	c.1061delG	Exon 8	Frameshift	**Novel**
**P17**	c.1117C>G (p.P373A)	Exon 8	Missense	**Novel**
**P18**	c.1174G>T (p.E392X)	Exon 8	Nonsense	Hessman, 1998, Cancer Res
**P19**	c.783+1 G>A	Intron 4	Splice site	Morelli,2000, Eur J Endocrinol
**P20**	c.313delC	Exon 2	Frameshift	**Novel**
**P21**	c.535 G>A (p.E179K)	Exon 3	Missense	Weinhaeusel,2000, Hum Mutat
**P22**	c.1049+1 G>T	Intron 7	Splice site	**Novel**
**P23**	c.912+2 T>C	Intron 6	Splice site	**Novel**
**P24**	c.196_200dupAGCCC	Exon 2	Frameshift	Park, 2003, Clin Genet
**P25**	c.228delC	Exon 2	Frameshift	Giraud,1998, Am J Hum Genet
**P26**	c.1350+1_1350+11del11	Intron 9	Splice site	Jager,2006, Mol Cell Endocrinol
**P27**	c.1350+1_1350+11del11	Intron 9	Splice site	Jager,2006, Mol Cell Endocrinol
**P28**	c.1308 G>A (p.W436X)	Exon 9	Nonsense	Crepin, 2003, Electrophoresis
**P29**	c.354_356delGAA	Exon 2	Frameshift	Bassett, 1998, Am J Hum Genet
**P30**	c.1579 C>T (p.R527X)	Exon 10	Nonsense	Lairmore, 2004, Ann Surg
**P31**	c.848 T>C (p.L283P)	Exon 6	Missense	**Novel**
**P32**	c.124 G>A (p.G42S)	Exon 2	Missense	Sato, 2000, Surgery
**P33**	c.912+1 G>A	Intron 2	Splice site	Mutch,1999, Hum Mutat

**Fig 1:**
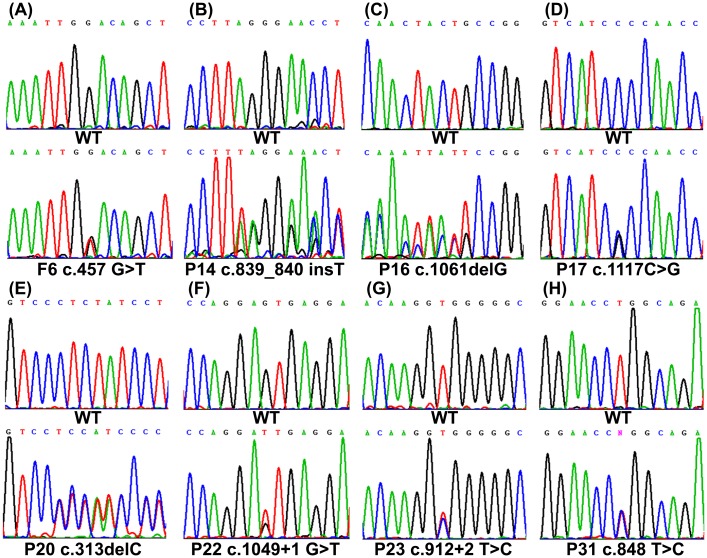
Direct sequencing of eight novel *MEN1* gene mutations. Sequencing showing 8 germline heterozygous point mutations in *MEN1* gene: (A) a missense mutation c.457 G>T (p.D153Y) in Family 6; (B) a frameshift mutation c.839_840 insT in Patient 14; (C) a frameshift mutation c.1061delG in Patient 16; (D) a missense mutation c.1117 C>G (p.P373A) in Patient 17; (E) a frameshift mutation c.313delC in Patient 20; (F) a splice site mutation c.1049+1 G>T in Patient 22; (G) a splice site mutation c.912+2 T>C in Patient 23; (H) a missense mutation c.848 T>C (p.L283P) in Patient 31.

**Fig 2:**
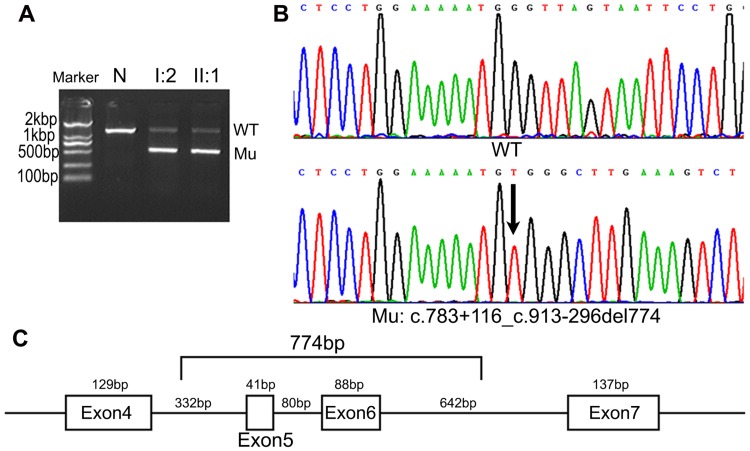
Direct sequencing of the gross deletion mutation of the *MEN1* gene in Family 3. (A) In order to find the exact deletion site of the gross deletion mutation detected by MLPA (*MEN1* del exon 5, 6), long-range PCR was used to amplify the region spanning exons 4–7 of the *MEN1* gene. Compared with the fragment (WT: 1330bp) generated from the normal control (N), the two patients of Family 3 (I:2 and II:1) both carried an aberrant fragment (Mu: approximately 500bp). (B) DNA sequence analysis of the mutant PCR product revealed that it was 774bp (the lower one) smaller than the normal controls (the upper one). (C) The deletion breakpoints were 116bp downstream of exon 4 and 296bp upstream of exon 7 (*MEN1* c.783+116_c.913-296del774).

Among all the mutations detected, nine mutations have not been previously reported. These novel mutations include *MEN1* c.783+116_c.913-296del774, c.457 G>T, c.839_840 insT, c.1061delG, c.1117C>G, c.313delC, c.1049+1 G>T, c.912+2 T>C, and c.848 T>C (Figs 1 and 2). One of these novel mutations was a gross deletion mutation detected by MLPA in one family. MLPA showed that the DQs of exon 5 and 6 were approximately equal to 50% in all patients from the family. Compared with normal controls, regular PCR revealed that these patients shared one aberrant fragment (approximately 500bp) that was shorter than the wild type (1330bp) (Fig 2A). Direct sequencing of the aberrant fragment confirmed the gross mutation as c.783+116_c.913-296del774 (Fig 2B and 2C).

None of these mutations were detected in the 100 normal reference individuals. Moreover, the nine novel mutations were all predicted to be “disease causing” by Polyphen-2, SIFT and Mutation Taster software.

### Immunohistochemical analysis

Marked nuclear expression of menin (scored as +++) was detected in tissues from normal parathyroid glands (S1 Fig). In contrast, specimens from 25 MHPT patients showed complete loss of menin expression in the nuclei (scored as -) (S1 Fig). Partial nuclear loss of menin (scored as ++) was observed in parathyroid samples from the other 6 MHPT patients (S1 Fig).

### Genotype-phenotype correlations

No significant differences were found between the patients in group 1 (with mutations resulting in truncated or partial loss of menin protein) and 2 (with missense mutations) regarding the clinical manifestations, biochemical parameters and pathology types (S2 Table).

## Discussion

PHPT in patients with MEN1 syndrome is associated with some unique features. To our knowledge, the present study is the largest clinical and genetic study concerning MEN1-related PHPT in a Chinese population. In addition, we supplemented the existing knowledge base with information on the clinical characteristics of MHPT in comparison to those of SHPT. The *MEN1* mutation rate was 79.4% in this study. Nine novel *MEN1* mutations, including one gross deletion mutation, were reported.

Our study demonstrated that MHPT patients exhibited characteristics of disease at a younger age along with milder biochemical presentation which were similar to those reported in Western countries [2, 3]. However, we also found some additional features that were different in MHPT patients compared with SHPT in our population. Lamers CB *et al* and Eller-Vainicher *et al* reported that the prevalence of kidney involvement was similar between MHPT and SHPT patients [3, 13]. However, urolithiasis was observed to be more common in our MHPT patients than in SHPT patients (60% vs. 40.2%). Although no significant difference in urinary calcium excretion was found in this study. Differences in sample size, ethnicity, and course of disease might have contributed to the inconsistencies between studies. Other descriptive studies performed only in MHPT patients have reported a similarly high frequency of renal calculi (57.8, 65%, and 75%)[3, 14, 15], supporting that urolithiasis is one of the frequent clinical manifestations in MHPT patients. Since the milder biochemical presentation does not align with the frequent occurrence of renal calculi in MHPT patients, the complications might be attributed to a different mechanism from that in PHPT with MEN1. Other factors, such as hyperoxaluria, hyperuricosuria and cystinuria, could also increase the risk of kidney nephrolithiasis. Therefore, further analysis of urinary biochemical stone risk profile is needed, which may help explain the high risk of kidney involvement in MHPT patients.

MHPT patients in our study showed milder biochemical presentation than SHPT patients, including lower levels of PTH and ALP with higher P level. Typical X-ray changes of PHPT were less common in our MHPT patients than SHPT patients (26.3% vs. 55.7%, P = 0.001), which aligned with the lower PTH and ALP levels observed in the MHPT group. MHPT patients could be diagnosed earlier than SHPT patients due to other MEN1 related endocrinopathies (such as insulinoma and pituitary functional adenoma). As showed in our data, asymptomatic PHPT was more common in the MHPT group (20.0%) than in the SHPT group (9.8%). Early diagnosis of MHPT could explain the milder X-ray changes in these patients. Regarding BMD detected by DXA, Eller-Vainicher *et al*. reported that MHPT patients show lower BMD of LS and FN than SHPT patients, even though MHPT patients exhibited milder biochemical presentation than their SHPT counterparts [3]. However we did not observed any significant differences in BMD of LS and FN between the two groups. Since results from X-ray and DXA did not align, further investigations employing new techniques, such as TBS and HR-pQCT, are required to evaluate the bone status between MHPT and SHPT patients. Accurate analysis may provide insights into the optimal timing for surgery in MHPT patients.

Furthermore, we found that SCa was not an independent risk factor for bone loss in MHPT patients, which is similar to the conclusion of Burgess [16]. These data indicated that mild hypercalcemia or normocalcemia could also be accompanied by reduced BMD, leading to higher risk of fracture. As recommended by Burgess, MHPT patients with elevated PTH greater than twice the upper limit of normal range should be considered as at risk for osteoporosis [16]. In our study, reduced BMD was more common in LS than in FN among MHPT patients (48.6% vs. 25.7%). In addition, the Z score of LS was lower than that of FN. Interestingly, the characteristics of bone loss in MHPT are different from those in SHPT. SHPT is characterized by bone loss at the cortical sites, and histomorphometric analyses and advanced imaging techniques have shown a relative preservation of cancellous bone [17, 18]. Previous investigations concerning BMD of MHPT also found that bone loss of LS was more severe than that of FN, suggesting that the relative protection of vertebral bone was not observed in MHPT patients [3, 15, 19]. Bone loss could be attributed to other endocrine dysfunctions such as hypercortisolism, hypogonadism, and GH deficiency caused by pituitary mass counteracting the anabolic effect of PTH on trabecular bone.

Twenty-seven germline *MEN1* mutations were detected in the present study with an overall mutation detection rate of 79.4%. PubMed and WanFang (a Chinese database) were utilized to summarize the results of genetic analysis in Chinese MEN1 patients. Most of the previous studies were case reports with the biggest sample size of 12 patients (6 sporadic and 6 familial cases) [20]. In all reported cases, 31 *MEN1* mutations were detected in 32 MEN1 families (96.9%) and 3 *MEN1* mutations were reported in 6 sporadic MEN1 patients (50%)[8, 9, 20–28]. Sporadic MEN1 cases were included in only one of these studies [20], which suggests that selection and publication bias might be present and therefore, real *MEN1* mutation rate could not be accurately calculated from these studies. Our study is the largest single-center research involving consecutive MHPT patients diagnosed from 2002 to 2013. The observed mutation detection rate in our population is similar to those reported in Caucasians [2] and in Japanese MEN1 cohort (75%)[29]. The *MEN1* mutation analysis is helpful for confirming the clinical diagnosis, suggesting early screening/treatment for tumors in family members with the *MEN1* mutation, and reducing the cost of screening for developing tumors in family members without the familial germline *MEN1* mutation [2]. Based on our results, we suggest that clinicians should consider the possibility of MEN1 among patients with relative endocrine tumors and utilize genetic testing for further diagnosis.

The majority of *MEN1* mutations detected in this study were inactivating mutations that lead to a truncated menin (74%), which occur at a similar rate as reported in published study (75%)[6]. In particular, a novel gross deletion (exon 5, 6) of *MEN1* gene was detected by MLPA in one of the families. Till now, 14 *MEN1* gross deletions have been reported, including 5 partial gene deletions and 9 whole gene deletions [6, 30–33]. Our result reinforces the current recommendation for screening exonic deletions using MLPA in patients, who match the clinical manifestation of MEN1 but with no mutations detected by direct DNA sequencing [2]. Our study was the first to screen for *CDKN1B* mutations, which have been shown to cause MEN4, in a Chinese population. Nevertheless, no *CDKN1B* mutation was detected in the present study, which supports the rarity of *CDKN1B* gene mutation.

The present study had some limitations. Firstly, due to the rarity of MEN1 in Chinese population, the sample size was limited. Secondly, the BMD of distal 1/3 radius could not be measured in our center due to the lack of normal reference and therefore, the non-classical symptoms was not evaluated due to the retrospective nature of this study. Thirdly, molecular function of novel mutations was not examined due to the lack of available assays that could be used to test for the pathogenetic effect of *MEN1* mutations [34]. Nevertheless, results from 100 normal individuals along with confirmation from *in silico* prediction software supported the pathogenicity of these mutations. Moreover, six novel mutations are inactivating mutations that lead to a truncated menin. Forth, the heterogeneity of studied subjects and lack of hotspot mutation region contributed to the difficulty in concluding any definite genotype-phenotype relationships. Fifth, SHPT was determined by negative family history and exclusion of MEN-1 related endocrine tumors because *MEN1* mutation screening was not routinely performed. Therefore, potential cases of rare isolated PHPT caused by *MEN1* mutation or late-onset MEN1 syndrome could not be fully excluded [35]. Finally, since 25-hydroxyvitamin D (25OHD) measurement was not routinely performed until recent years, the available data on 25OHD were insufficient for statistical analysis in this retrospective study.

## Conclusion

In summary, this is the largest study describing the clinical characteristic and genetic background of MHPT in a Chinese population. Compared with their SHPT counterparts, MHPT patients experienced more common kidney complications but less common skeletal involvement along with a milder biochemical presentation. In order to formulate more reasonable management strategies, such as the optimum timing for parathyroidectomy in MHPT, future investigation on comparing the clinical outcomes of MHPT and SHPT patients and data from long-term follow-up are much needed. Our study also highlighted the potential of using MLPA as a complementary test to address some of the deficiencies of traditional sequencing.

## Supporting Information

S1 Fig IHC staining of menin in referred patients parathyroid tumors (A-B) and normal parathyroid(C). (DOCX)

S1 Table Primer used for ordinary PCR amplification of the *MEN1* gene and *CDKN1B* gene. (DOCX)

S2 Table Comparision between the MHPT patients with and without truncated *MEN1* mutations. (DOCX)
