# Supplementary material for: Clinical and Genetic Analysis of Multiple Endocrine Neoplasia Type 1-Related Primary Hyperparathyroidism in Chinese
Source: PLoS One. 2016 Nov 15;11(11):e0166634. doi: 10.1371/journal.pone.0166634 (PMC5112846; doi:10.1371/journal.pone.0166634)

**S1 Fig.** **IHC staining of MENIN in referred patients parathyroid tumors (A-B) and normal parathyroid(C).** All images are magnified 400X. (A) and (B) were the [specimens](app:ds:specimens) of MEN1-related parathyroid tumors. (C) was the specimen of normal parathyroid. (D) The primary antibody was replaced with water used as negative control. (A) showed nuclear loss of MENIN completely. (B) showed positive staining within less than 95% in nuclei, indicating partial nuclear loss of MENIN. (C) Normal parathyroid gland demonstrated strong nuclear staining for MENIN (>95%).


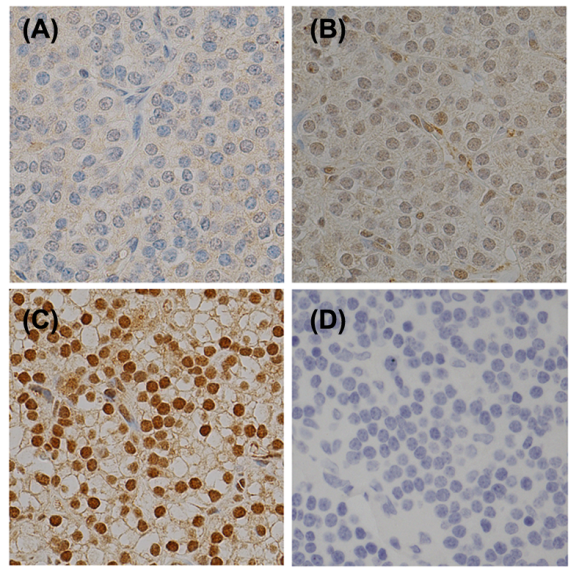

Supplement: S1 Fig — (DOCX) [file pone.0166634.s001.docx]
