# Supplementary material for: Clinical and Genetic Analysis of Multiple Endocrine Neoplasia Type 1-Related Primary Hyperparathyroidism in Chinese
Source: PLoS One. 2016 Nov 15;11(11):e0166634. doi: 10.1371/journal.pone.0166634 (PMC5112846; doi:10.1371/journal.pone.0166634)
Supplement: S1 Table — (DOCX) [file pone.0166634.s002.docx]

**S1 Table.** Primer used for ordinary PCR amplification of the *MEN1* gene and *CDKN1B* gene.

| **Gene** | **Exon** | **Forward primer（F）** | **Reverse primer（R）** | **Size**  **(bp)** |
| --- | --- | --- | --- | --- |
| ***MEN1*** | **2** | TGTCGGGGCGGGTGGAACCTTA | GCCGAACCTCACAAGGCTTACAGT | 660 |
|  | **3-4** | AGAATCTGAGGTTGGGTCAC | GGCCAGGAATTACTAACCCAT | 805 |
|  | **5-6** | GTCCCTGTTGGTTCTGACCCC | GATTCTGCACACAGTTGACACA | 450 |
|  | **7-8** | GATCTTCCTGTGGCCCCTT | TCCTGCCATCCCTAATCCCGTA | 938 |
|  | **9** | TAGAGGTTTCTACCCTGTGCCTT | GTCTCAGTCCCATCGGCACC | 538 |
|  | **10** | CCACGGGCTTGTCAGACT | TACTCGGGACCGGGAACCTA | 788 |
|  | **4-7** | ATCAACCCTTCCATTGACCTG | CACATTGCGGTTGCGACA | 1330 |
| ***CDKN1B*** | **1** | TCTGTGTCTTTTGGCTCCGA | AGCACTGAACACCTAAGACCA | 729 |
|  | **2** | TTTGTGCCCTTAAAAGCCACT | CCAGCAACCAGTAAGATCAGG | 364 |

**Annealing temperature in PCR used:**

Exon 2 of the *MEN1* gene:

The annealing temperature of the reaction is decreased 0.5℃ every second cycle from 70℃ to a “touchdown” at 60℃, at which temperature 20 cycles are carried out.

Exon 3-4, 5-6, 7-8, 9, 10 of the *MEN1* gene:

The annealing temperature of the reaction is decreased 0.5℃ every second cycle from 65℃ to a “touchdown” at 55℃, at which temperature 20 cycles are carried out.

Exon 4-7 of the *MEN1* gene: 58℃

Exon 1 and 2 of the *CDKN1B* gene: 57℃
