# Supplementary material for: Clinical and Genetic Analysis of Multiple Endocrine Neoplasia Type 1-Related Primary Hyperparathyroidism in Chinese
Source: PLoS One. 2016 Nov 15;11(11):e0166634. doi: 10.1371/journal.pone.0166634 (PMC5112846; doi:10.1371/journal.pone.0166634)
Supplement: S2 Table — (DOCX) [file pone.0166634.s003.docx]

**S2 Table.** Comparision between the MHPT patients with and without truncated *MEN1* mutations.

|  | MHPT Group1  (n=25) | MHPT Group2  (n=8) | P | P^#^ |
| --- | --- | --- | --- | --- |
| Sex (M/F) | 7/18 | 4/4 | 0.391 |  |
| Age^*^ (years) | 42.2±12.2 | 53.2±14.4 | 0.059 |  |
| Course of PHPT (years) | 7 (8) | 7(25) | 0.471 |  |
| Typical changes in X-rays^§^ | 29.2%(7/24) | 25%(2/8) | 1.000 | 0.537 |
| Gastrointestinal symptoms | 36%(9/25) | 12.5%(1/8) | 0.382 | 0.162 |
| Urolithiasis/renal calcification | 48%(12/25) | 75%(6/8) | 0.242 | 0.134 |
| PNET | 24/24(100%) | 7/7(100%) | / | / |
| Pituitary adenoma | 20/24(83.3%) | 5/8(62.5%) | 0.327 | 0.787 |
| SCa (mmol/L) | 2.89±0.23 | 2.88±0.24 | 0.926 | 0.775 |
| iCa (mmol/L) | 1.41±0.19 | 1.42±0.14 | 0.917 | 0.826 |
| P (mmol/L) | 0.82±0.14 | 0.82±0.15 | 0.956 | 0.665 |
| ALP (U/L) | 120.0(100.0) | 73.5(51.8) | 0.029 | 0.803 |
| PTH (×UL ) | 4.5(4.7) | 6.3(5.8) | 0.588 | 0.700 |
| UCa (mmol/24h) | 9.12±4.17 | 10.97±4.12 | 0.285 | 0.404 |
| UP (mmol/24h) | 18.60±7.34 | 19.18±6.14 | 0.852 | 0.827 |
| LS BMD(Z-score) | -1.907±1.359 | -1.327±1.728 | 0.339 | 0.616 |
| FN BMD(Z-score) | -1.708±1.323 | -1.621±0.984 | 0.867 | 0.655 |
| LS BMD (T-score) | -2.338±1.447 | -2.046±1.691 | 0.642 | 0.485 |
| FN BMD (T-score) | -1.931±0.907 | -2.202±0.473 | 0.429 | 0.464 |

Group 1: MHPT patients carried nonsense, frameshift, gross deletion, and splice site mutations of *MEN1* gene. Group 2: MHPT patients carried missense mutations of *MEN1* gene.

*, the age at diagnosis of PHPT. #, P value adjusted for age, sex and course of PHPT. Data are expressed as mean ± SD, median (inter-quartile range) or as percentage. §, typical changes of PHPT in X-ray including subperiosteal absorption, osteitis fibrosa cystic and osteomalacia.

PHPT: primary hyperparathyroidism. MHPT: multiple endocrine neoplasia type 1-related PHPT. PNET: pancreatic neuroendocrine tumor. F: female; M: male; SCa: serum total calcium. iCa: serum ionized calcium. P, serum phosphorous. ALP, alkaline phosphatase. PTH, serum intact parathyroid hormone. UL: upper limit. Cr, creatinine. UCa, 24h urinary calcium. UP, 24h urinary phosphorous. BMD, bone mineral density. LS, lumbar spine. FN, femoral neck.
